# Supplementary material for: A fault-intrusion-tolerant system and deadline-aware algorithm for scheduling scientific workflow in the cloud
Source: PeerJ Comput Sci. 2021 Nov 2;7:e747. doi: 10.7717/peerj-cs.747 (PMC8576554; doi:10.7717/peerj-cs.747)
Supplement: Supplemental Information 6 [file peerj-cs-07-747-s006.zip › Myproject1.1/docs/org/cloudbus/cloudsim/network/TopologicalNode.html]

TopologicalNode


JavaScript is disabled on your browser.


- Overview
- Package
- Class
- Use
- Tree
- Deprecated
- Index
- Help

- Prev Class
- Next Class

- Frames
- No Frames

- All Classes

- Summary:
- Nested |
- Field |
- Constr |
- Method

- Detail:
- Field |
- Constr |
- Method


org.cloudbus.cloudsim.network

## Class TopologicalNode

- java.lang.Object
- - org.cloudbus.cloudsim.network.TopologicalNode

- ---

    

  ```
  public class TopologicalNode
  extends java.lang.Object
  ```

  Just represents an topological network node retrieves its information from an
  topological-generated file (eg. topology-generator)

  Since:
  :   CloudSim Toolkit 1.0

- - ### Constructor Summary

    Constructors

    Constructor and Description

    `TopologicalNode(int nodeID)`

    constructs an new node

    `TopologicalNode(int nodeID,
    int x,
    int y)`

    constructs an new node including world-coordinates

    `TopologicalNode(int nodeID,
    java.lang.String nodeName,
    int x,
    int y)`

    constructs an new node including world-coordinates and the nodeName
  - ### Method Summary

    Methods

    | Modifier and Type | Method and Description |
    | --- | --- |
    | `int` | `getCoordinateX()` returns the x coordinate of this network-node |
    | `int` | `getCoordinateY()` returns the y coordinate of this network-node |
    | `int` | `getNodeID()` delivers the nodes id |
    | `java.lang.String` | `getNodeLabel()` delivers the name of the node |

    - ### Methods inherited from class java.lang.Object

      `clone, equals, finalize, getClass, hashCode, notify, notifyAll, toString, wait, wait, wait`

- - ### Constructor Detail


    - #### TopologicalNode

      ```
      public TopologicalNode(int nodeID)
      ```

      constructs an new node


    - #### TopologicalNode

      ```
      public TopologicalNode(int nodeID,
                     int x,
                     int y)
      ```

      constructs an new node including world-coordinates


    - #### TopologicalNode

      ```
      public TopologicalNode(int nodeID,
                     java.lang.String nodeName,
                     int x,
                     int y)
      ```

      constructs an new node including world-coordinates and the nodeName
  - ### Method Detail


    - #### getNodeID

      ```
      public int getNodeID()
      ```

      delivers the nodes id

      Returns:
      :   just the nodeID


    - #### getNodeLabel

      ```
      public java.lang.String getNodeLabel()
      ```

      delivers the name of the node

      Returns:
      :   name of the node


    - #### getCoordinateX

      ```
      public int getCoordinateX()
      ```

      returns the x coordinate of this network-node

      Returns:
      :   the x coordinate


    - #### getCoordinateY

      ```
      public int getCoordinateY()
      ```

      returns the y coordinate of this network-node

      Returns:
      :   the y coordinate


- Overview
- Package
- Class
- Use
- Tree
- Deprecated
- Index
- Help

- Prev Class
- Next Class

- Frames
- No Frames

- All Classes

- Summary:
- Nested |
- Field |
- Constr |
- Method

- Detail:
- Field |
- Constr |
- Method
